# Food-Based Therapies in Pediatric Inflammatory Bowel Diseases

**DOI:** 10.3390/nu18152493

**Published:** 2026-08-02

**Authors:** Irene Dalpiaz, Luca Scarallo, Alessia De Blasi, Margherita Vita, Benedetta Pasquini, Elena Banci, Sara Renzo, Elena Sofia Pieri, Giulia D’Arcangelo, Salvatore Oliva, Fabio Salvatore Macaluso, Angelo Campanozzi, Paolo Lionetti

**Affiliations:** 1 Gastroenterology and Nutrition Unit, Meyer Children’s Hospital IRCCS, 50139 Florence, Italy; 2 Department of Chemistry “Ugo Schiff”, University of Florence, Sesto Fiorentino, 50019 Florence, Italy; 3 Pediatrics, Department of Medical and Surgical Sciences, University of Foggia, 71122 Foggia, Italy; 4 Gastroenterology, Hepatology and Cystic Fibrosis Unit, Fondazione IRCCS Cà Granda, Ospedale Maggiore Policlinico di Milano, 20122 Milan, Italy; 5 Pediatric Gastroenterology and Liver Unit, Department of Maternal Infantile and Urological Sciences, Sapienza University of Rome, 00161 Rome, Italy; 6 IBD Unit, “Villa Sofia-Cervello” Hospital, 90146 Palermo, Italy; 7 Department NEUROFARBA, University of Florence, 50139 Florence, Italy

**Keywords:** Crohn’s disease, ulcerative colitis, Pediatrics, Crohn’s disease exclusion diet, Mediterranean diet, Western diet, nutrition, dietary management

## Abstract

The incidence of inflammatory bowel diseases is rising both in adults and children, particularly in regions traditionally considered low-incidence, in parallel with the increasing adoption of Westernized lifestyles, especially dietary habits. Growing evidence implicates harmful dietary patterns, such as the Western diet, in the development and persistence of IBD, fueling interest in dietary modulation as a therapeutic strategy to promote disease remission. Beginning with the exclusive enteral nutrition as an induction therapy for Crohn’s disease (CD), several nutritional regimens have been proposed to improve treatment tolerability and adherence by introducing whole-food-based approaches. To date, various food-based therapies focused on excluding potentially pro-inflammatory components and incorporating foods with potential anti-inflammatory components have been explored, mainly in pediatric CD, while their application in ulcerative colitis remains largely experimental. In this narrative review, we summarize the food-based therapeutic approaches proposed for pediatric IBD, discussing both the rationale underlying their use and the current evidence supporting their implementation.

## 1. Introduction

Inflammatory bowel diseases (IBDs), including ulcerative colitis (UC), Crohn’s disease (CD), and IBD-unclassified, are chronic immune-mediated diseases developing during childhood in about 10% to 20% of cases [1,2,3,4].

Although several genetic and environmental factors influencing IBD pathogenesis have been identified, the exact mechanisms underlying these diseases remain unclear. Genome-wide association studies have uncovered more than 200 IBD-associated loci accounting for only approximately 25% of IBD inheritability [5,6]. Environmental factors likely play a critical role in disease development in genetically susceptible individuals. Among these factors, accumulating evidence highlights the substantial impact of diet on both the onset and progression of IBD. This is supported by the sharp rise in incidence of IBD in regions traditionally considered low risk, such as Southern Europe, Asia, and developing countries, where Western habits are increasingly adopted [7,8,9,10]. In particular, the Western diet (WD), characterized by high consumption of fatty acids, animal proteins, and refined sugars, alongside low intake of fruits and vegetables, has emerged as a key contributor to IBD [11]. Dietary components of the WD may promote and maintain intestinal inflammation by altering the intestinal microbiota, disrupting the mucosal barrier function, and impairing the local immune responses [12]. Further supporting the influence of environment and Westernized lifestyle patterns, migration studies have demonstrated an increased risk of IBD among individuals moving from low-incidence to high-prevalence regions, especially among first-generation children [13,14].

Despite growing evidence that diet contributes to both the development and the persistence of IBD, current guidelines still emphasize pharmacological approaches focused on immune suppression, such as corticosteroids, thiopurines, and biologic therapies [15,16]. In recent years, increasing attention has been directed toward the role of diet in the management of IBD, particularly in CD, whereas food-based therapies for UC remain mostly experimental. The role of nutritional therapy in CD has become well established, with exclusive enteral nutrition (EEN), involving the use of a complete liquid formula as the sole source of food for 6 to 8 weeks, currently recommended by international guidelines for CD management as induction therapy [15].

Although the efficacy of EEN has been extensively demonstrated in the literature, showing comparable rates of clinical remission to corticosteroids and higher rates of mucosa healing [17,18], this nutritional intervention is often associated with poor tolerance and lack of adherence among patients. Building on these limitations, recent research has focused on food-based therapies to induce and maintain remission.

In this narrative review, we aim to summarize the food-based therapeutic approaches currently proposed for pediatric IBD, discussing both the rationale underlying their use and the available evidence supporting their implementation, which is further summarized in Table 1, reporting original studies included in this review. These approaches generally rely on two key principles: the exclusion of potentially harmful foods and the inclusion of foods with putative beneficial effects, with the ultimate goal of reducing intestinal inflammation. As some dietary strategies have been evaluated exclusively in adult populations, references to these studies will also be included to highlight potential directions for future pediatric research.

## 2. Diet and IBD Pathogenesis

IBDs are multifactorial diseases involving genetic, environmental, microbial, and immunological factors in their pathogenesis. Although specific triggers remain unclear, diet appears to play a crucial role in disease development and progression.

Nutritional habits, particularly the WD, have been proposed as a major environmental factor for non-communicable diseases, including IBDs [51]. The WD is characterized by a high intake of calorie-dense processed foods, saturated fats, salt, and refined sugars, along with a low consumption of fruits and vegetables, and has been consistently associated with a pro-inflammatory state. Notably, Temba et al. reported significant immune and metabolic alterations linked to WD in a randomized controlled trial (RCT) assessing the effects of a 2-week dietary switch between heritage-style diet, which is predominantly plant-based, rich in fiber and polyphenols, and includes a fermented banana beverage made with finger millet, namely the “Mbege”, and Western dietary habits. Participants transitioning from the WD to the heritage diet exhibited anti-inflammatory changes. Conversely, those switching from heritage diet to the WD showed multiple pro-inflammatory responses, including increased levels of inflammatory proteins; reduced concentrations of anti-inflammatory metabolites, such as eicosapentaenoic acid and docosahexaenoic acid; upregulation in immune-related genes; and alterations in leukocyte number and phenotype, likely driven by enhanced myelopoiesis and immune cell activation [52].

Furthermore, several studies have explored the role of the WD in IBD onset and flares [53,54,55]. WD is associated with specific microbiota shifts, including an increase in mucin-degrading bacteria, such as *Bacteroides* and *Akkermansia* species, *Proteobacteria*, and adherent-invasive *Escherichia coli*, alongside a reduction in short chain fatty acid (SCFA)-producing bacteria [56,57,58]. Notably, in a large prospective cohort study including 125,445 participants followed for up to 14 years, Peters et al. observed 224 new UC and 97 new CD cases. Their study demonstrated that adherence to a Western dietary pattern was associated with a higher risk of UC (odds ratio [OR]: 1.16, 95% confidence interval [CI]: 1.03–1.30, *p* = 0.013; OR: 1.11, 95%CI: 1.01–1.20, *p* = 0.023, respectively) [59]. Similarly, Guo et al. evidenced a role of diet in IBD’s development, following 81,280 individuals from birth through childhood and adolescence. The authors reported 307 new IBDs, and while assessing dietary habits, they observed that a high intake of fish, vegetables, and fruits in early life was associated with a lower risk of IBD (pooled hazard ratio [HR] for fish: 0.66, 95%CI: 0.46–0.93; HR for vegetables and fruits: 0.72, 95%CI: 0.55–0.95). Conversely, early and high consumption of sugary drinks was associated with an increased risk of IBD (pooled HR: 1.42, 95%CI: 1.05–1.90) [60].

Focusing on individual WD components, a systematic review by Hou et al. demonstrated that high dietary intake of total fats, PUFAs, omega-6 fatty acids, and meat was associated with increased risk of CD and UC, whereas high intake of fiber and fruit reduced risk of CD, and high vegetable intake reduced UC risk [11]. The impact of ultra-processed foods has also been investigated, reporting a positive association with IBD development, particularly CD [61,62,63]. Finally, a large prospective cohort study on 245,112 participants documented 369 incident cases of CD and 488 incident cases of UC and described an association between higher ultra-processed foods and CD (HR: 1.70; 95%CI: 1.23–2.35; *p*-value < 0.05), although without a consistent association with UC [64]. Similarly, high consumption of red and processed meat, alcohol, and foods and beverages rich in sulfur and sulfate was associated with an increased risk of UC [65]. Red and processed meat, in particular, appear to contribute to IBD pathogenesis by fostering dysbiosis, with reduction in beneficial species, such as *Streptococcus*, *Akkermansia*, *Faecalibacterium*, and *Lactococcus*, and increases in *Clostridium* and *Mucispirillum* species [66,67]. Moreover, a high consumption of meat may contribute to disease flares in patients with UC, as demonstrated in the PREdiCCt study, underscoring the importance of dietary habits not only in IBD pathogenesis but also in disease course [68].

An emerging factor in IBD pathogenesis is the role of SCFAs, bacterial fermentation products of dietary fiber fermentation, mainly represented by butyrate, propionate, and acetate. Evidence supports an anti-inflammatory role for SCFAs, as they enhance epithelial barrier integrity, regulate immune responses, and serve as a critical energy source for colonocytes [69]. Consistently, patients with IBD show reduced levels of SCFAs due to dysbiosis and a decrease in SCFA-producing bacteria [70,71]. This reduction, alongside the promotion of gut inflammation, highlights that SCFAs may represent a potential therapeutic target [72,73].

Diet also modulates immune responses, as nutrients with anti-inflammatory potential, such as ω-3, polyunsaturated fatty acids (PUFAs), carotenoids, flavonoids, terpenes, vitamins, and fibers, have been shown to reduce the production of pro-inflammatory mediators, such as interleukin-6, interleukin-1β, interleukin-8, tumor necrosis factor-α, reactive oxygen species, nitric oxide, and prostaglandins [74].

Given the established influence of diet on IBD, it is reasonable to consider diet as a therapeutic strategy, aiming to minimize exposure to pro-inflammatory ingredients, characteristics of the WD, and to increase the intake of anti-inflammatory ingredients. At the same time, nutritional therapies may reduce the risk of adverse effects typically associated with immune-targeting pharmacological treatments. Strong initial evidence supporting diet-based interventions includes data demonstrating the efficacy and safety of EEN in inducing remission in CD [75,76,77,78]. However, EEN faces important barriers, most notably the monotony of the regimen and the taste fatigue experienced by patients [79]. These factors substantially limit the accessibility and acceptability of this therapeutic option [80]. Furthermore, it has been acknowledged that a rapid re-exacerbation of inflammation, demonstrated by a rise in fecal calprotectin (FC), often occurs within 2–3 weeks after completing an EEN course and reintroducing whole foods. Maintenance enteral nutrition has likewise proven ineffective for sustaining long-term remission [81]. Hence, re-establishment of a normal diet frequently leads to renewed intestinal inflammation [82]. This context underscores a significant unmet need: understanding how dietary manipulation can maintain remission. Consequently, in recent years, researchers have sought to replicate the therapeutic benefits of EEN using dietary strategies that permit the consumption of whole foods.

## 3. Food-Based Therapies

### 3.1. Crohn’s Disease Exclusion Diet (CDED)

The CDED, which was first described in 2014 by Sigall-Boneh et al., based on the hypothesis that partial enteral nutrition (PEN), combined with the exclusion of specific dietary components, may modulate the intestinal microbiome and permeability, thereby promoting improvement in CD [22].

CDED is a standardized, high-protein, low-fat dietary regimen structured in three phases, progressing from a more restrictive phase to a more liberated diet. It is based on the exclusion of potentially harmful foods, including processed products, animal fats, gluten, and dairy, as well as additives such as emulsifiers, maltodextrins, carrageenan, and sulphites. Guided by the principles of exclusion and inclusion, natural foods are combined with a variable proportion of polymeric formula to meet the nutritional requirements and ensure adequate intake of protein, calcium, and vitamin D. Phase 1, during at least 6 weeks, combines CDED with PEN, providing 50% of the nutritional requirement. This phase is the most restrictive, eliminating pro-inflammatory components and limiting fruit and vegetable intake. Five mandatory foods, namely apples, bananas, chicken, eggs, and potatoes, are also scheduled in phase 1. Phase 2, which also lasts approximately 6 weeks, allows for gradual dietary expansion, including increased fiber intake and controlled reintroduction of potentially noxious foods, such as gluten and red meat. In phase 2, PEN is reduced to 25% of caloric needs [83]. Phase 3, lasting at least 9 months, promotes mucosa healing in children and adolescent who achieved clinical remission during the first 12 weeks. PEN continues to provide 25% of caloric needs, with a broad, whole-food diet emphasizing fruit and vegetable intake. Ultra-processed foods and items high in saturated fatty acids and refined sugars remain excluded.

Summarizing the rational underlying CDED, Levine et al. proposed the hypothetical “bacterial penetration cycle model”: exposure of the intestinal mucosa to harmful dietary components may induce dysbiosis and favor the selection of pro-inflammatory bacterial species, leading to inflammation, disruption of the intestinal barrier, and enhanced translocation of pathogenic bacteria, thus perpetuating a vicious cycle of mucosal damage and immune activation [84]. Supporting this hypothesis, the same authors reported changes associated with clinical remission, including reductions in *Haemophilus*, *Veillonella*, *Anaerostipes,* and *Prevotella*, alongside increases in *Roseburia* and *Oscillibacter*. Interestingly, a sustained reduction in *Proteobacteria* was observed in children treated with CDED plus PEN, a feature associated with corticosteroid-free remission [19]. Furthermore, Verburgt et al. investigated the impact of both CDED with PEN and EEN on gut microbiota, showing that by week 12, microbial composition in both groups approached that of healthy controls, particularly with a decrease in the relative abundance of *Proteobacteria*. These diet-induced microbial shifts are thought to promote the expansion of beneficial taxa potentially linked to clinical remission and mucosal healing [20]. Notably, PEN may exert beneficial effects on the gut microbiota, potentially through a synergistic effect with CDED. This is supported by the findings from Rohani et al., who conducted an RCT demonstrating that children receiving CDED with PEN showed greater increase in *Bifidobacterium* and *Lactobacillus,* along with a more pronounced reduction in *E. coli* and *Fusobacterium,* compared with those receiving CDED alone [21].

In the first study describing CDED, Sigall-Boneh et al. demonstrated high remission rates in children with early mild-to-moderate luminal CD, reporting clinical response and remission rates of 78.7% and 70.2%, respectively [22]. Building on these findings, subsequent studies further confirmed the efficacy of CDED. Niseteo et al. found comparable efficacy between CDED with PEN and EEN, with remission rates of 75.0% and 68.9%, respectively, and significantly greater weight gain and increases in BMI z-scores in the CDED group (*p* = 0.002 and *p* = 0.001, respectively) [23]. Moreover, a randomized controlled trial (RCT) in children and adolescents with mild-to-moderate CD showed a higher rate of sustained remission among those receiving CDED with PEN compared to EEN [19]. A more recent RCT conducted by Sigall Boneh et al. in pediatric patients with mild-to-severe CD demonstrated maintenance of remission up to 24 weeks in 60% of participants, although the study was meant to establish superiority over EEN [24].

Evidence also supports the use of CDED combined with PEN as an effective salvage regimen for patients who failed biologic therapy [25,26]. In a real-world study, Scarallo et al. confirmed the efficacy of CDED with PEN in children and adolescents with refractory and severe disease, though patients with extraintestinal manifestations showed lower clinical response rates (*p* = 0.018) [27]. Similarly, Jijón Andrade, in a retrospective study, reported a good remission rate in children and adolescents relapsing on biologic therapy (60%) [28]. Furthermore, CDED with PEN may play a role in enhancing and accelerating the efficacy of anti-TNFα agents in inducing both clinical and biochemical remission in severe disease setting, as suggested by Pochesci et al. In their study, the authors compared biochemical and clinical outcomes in children with CD treated with CDED with PEN in combination with anti-TNFα agents compared to those receiving biologic therapy alone. The combined approach was associated with a higher rate of normalization of erythrocyte sedimentation rate and CRP normalization at 8 weeks (*p* = 0.027 and *p* = 0.050, respectively), as well as a greater improvement in clinical activity, assessed by the weighted Pediatric Crohn’s Disease Activity Index (PCDAI) (*p* = 0.001) [29].

Despite robust evidence for clinical efficacy, data on endoscopic outcomes remain limited. In a prospective cohort of children with active CD, Urlep et al. observed similar endoscopic response and remission rates at week 6 for EEN and CDED (modified for local dietary customs) with PEN: 68.8% vs. 84.6% (*p* = 0.41) and 50.0% vs. 53.8% (*p* = 0.99) [30]. Similarly, Martìn-Masot assessed mucosal inflammation using the Mucosal Inflammation Non-Invasive (MINI) index, which reliably reflects mucosa healing, and found a significant improvement from baseline to 52 weeks (*p* < 0.05) [31].

Although no extensively investigated, CDED with PEN appears to have a beneficial effect on bone metabolism, in contrast to corticosteroids, which are known to impair bone density [85,86]. Lev-Tzion et al. assessed markers of bone formation, such as C-Propeptide of Type I Procollagen, and bone mineral density via dual energy X-ray absorptiometry in children receiving either CDED with PEN or EEN during over 24 weeks. Both groups showed significant improvement in C-Propeptide of Type I Procollagen levels, though no significant changes in bone mineral density were observed [32].

Given its demonstrated efficacy and improved tolerability, CDED with PEN represents a valuable alternative to EEN, which is often poorly tolerated by children and adolescents [33]. In a study by Levine et al., tolerance to CDED with PEN was significantly better than to EEN (97.5% vs. 73.6%, *p* = 0.002) [19]. Moreover, real-life data from a Dutch cohort of children and adults with mild-to-moderate CD showed a good experience with CDED with PEN in most patients: 83% of the study sample would recommend CDED to others, while two-thirds of them would reconsider starting CDED again [33]. Nevertheless, adherence challenges remain due to dietary restrictions. A study conducted in Saudi Arabia found that only 57% of the patients continued the diet beyond 12 weeks, with poor compliance attributed to intolerance, difficulty following the regimen, costs, and limited clinical response [34].

### 3.2. Specific Carbohydrate Diet and Modified Specific Carbohydrate Diet (SCD)

The SCD is an exclusionary dietary regimen first developed in the 1930s as a therapeutic approach for celiac disease, and more recently proposed as a potential intervention for IBD. The SCD eliminates all grains and sweeteners except for honey. Milk and milk-derived products are restricted, except for hard cheeses and yogurt fermented for greater than 24 h. In addition, food additives such as emulsifiers, commonly found in processed foods, are excluded from the diet [35]. While the SCD may provide adequate nutrient intake, close nutritional monitoring is recommended, as supplementation with vitamin D may be necessary to prevent deficiencies [87]. A less restrictive modification of the SCD, referred to the modified SCD (mSCD), has been proposed, allowing limited inclusion of oats and rice [88].

Current evidence on SCD and mSCD in pediatric IBD remains limited and is primarily derived from case reports and small case series, while most studies have been conducted in adults. Suskind et al. reported clinical improvement in seven children with CD treated exclusively with the SCD and no concomitant pharmacological therapy. Over a follow-up period ranging from 5 to 30 months, patients showed improvements in both clinical symptoms and laboratory parameters, including serum albumin, CRP, hematocrit, and FC [35]. Similarly, Obih et al. observed significant clinical and biochemical improvement in children with CD receiving the SCD, with a reduction in the PCDAI, which decreased from 32.8 ± 13.2 o 20.8 ± 16.6 at week 4, and to 8.8 ± 8.5 by 6 months [36]. The broader mSCD variant has also been reported to reduce clinical symptoms and systemic inflammation, as reflected by decrease in CRP [88].

Evidence regarding the endoscopic impact of SCD is also scarce. Wahbeh et al. evaluated mucosal healing, defined as the absence of ulceration from the upper gastrointestinal and ileocolonic tracts, in seven children with CD following the mSCD, and found no cases of complete mucosal healing [37]. Similarly, Britto et al. described three pediatric CD patients who achieved durable clinical remission with normalization of FC on strict SCD monotherapy, but without evidence of mucosal healing [38]. In contrast, Cohen et al. reported both clinical and endoscopic improvements after a 12-week SCD intervention in nine children with active CD, as demonstrated by reductions in PCDAI (*p* = 0.011) and Lewis score (*p* = 0.012) on capsule endoscopy [39]. Supporting these findings, Simon et al. documented a case of sustained clinical, biochemical, radiographic, endoscopic, and histologic remission in a pediatric patient with penetrating CD with SCD monotherapy [40].

### 3.3. Crohn’s Disease Treatment-with-EATing (CD-TREAT)

CD-TREAT is a food-based therapeutic approach introduced in 2019 as an alternative to EEN. It aims to replicate the composition and anti-inflammatory properties of EEN by excluding potentially pro-inflammatory ingredients, such as dairy products, gluten, and alcohol. The diet is designed to provide adequate amounts of macronutrients, vitamins, minerals, and dietary fibers, while maintaining similarity to a regular diet to enhance palatability and adherence.

The only RCT evaluating CD-TREAT included 25 healthy adults and five children with active CD. Among the pediatric participants, clinical response was achieved in four of five children, with clinical remission in three of five, accompanied by a significant reduction in FC (*p* = 0.002) [41]. In agreement with these findings, Svolos et al. conducted an open-label study involving 25 children and 32 adults with CD, which demonstrated encouraging results. After 8 weeks of CD-TREAT, the majority of participants exhibited improvements in both disease activity and quality of life (QoL). In the pediatric cohort, QoL assessed using the IMPACT-III questionnaire significantly increased from a baseline score of 136 (interquartile range: 22–143) to 148 (interquartile range: 133–153) at week 8 (*p* < 0.01) [42].

### 3.4. Tasty and Healthy (T&H) Dietary Approach

The T&H Dietary Approach is a whole-food diet characterized by the exclusion of pro-inflammatory and processed foods, including industrialized foods, animal fats, and gluten. It does not rely on specific formulas or mandatory ingredients. This food-based therapy encourages the consumption of fresh, unprocessed foods, such as vegetables, fruits, legumes, rice, gluten-free grains, eggs, poultry, seafood, and fish [89].

First, the TASTI-MM RCT evaluated the efficacy of the T&H diet in 83 biologic-naive patients aged 6–25 years with mild-to-moderate CD comparing T&H with EEN. Patients were randomized to T&H or EEN for 8 weeks, and tolerability, symptomatic remission, MINI index, FC, CRP, and erythrocyte sedimentation rate (ESR) were evaluated. The RCT demonstrated better tolerability of T&H compared to EEN (88% vs. 52%, *p* < 0.001) and higher remission rates, assessed by the weighted PCDAI and CD activity index (CDAI) (56% vs. 38%, *p* = 0.09). Importantly, only mild and infrequent adverse events were reported in the T&H group, with only one patient experiencing stypsis. Furthermore, the RCT revealed a beneficial modulation of the gut microbiota associated with T&H, showing superior species richness at both week 4 and week 8. Specifically, patients receiving the T&G but not the EEN exhibited a significant reduction of pro-inflammatory bacteria species such as *Ruminococcus gnavus* (*p* < 0.001). A higher abundance of *Faecalibacterium prausnitzii* and *Bacteroides uniformis*, two species associated with intestinal healing, was also observed in the T&H group compared to the EEN one (*p* = 0.002 and *p* = 0.006, respectively, for the two species) [43]. Plotkin et al. further supported the effectiveness of the T&H diet in the TAST-E RCT, involving pediatric and adult patients with CD who were in clinical remission or with minimal symptoms. Participants were assigned either to receive the T&H diet or to continue their habitual diet for 8 weeks, after which the latter group also initiated the T&H intervention. The authors reported a significantly higher FC response, defined as a reduction > 50%, in the T&H group compared to the habitual diet group (37% vs. 15%, *p* = 0.028). This was accompanied by a greater improvement in CRP levels and MINI index scores (*p* = 0.001 and *p* = 0.047, respectively). Notably, high adherence to the T&H diet was observed throughout the study [44].

The use of the T&H diet as maintenance treatment has recently been investigated in the MyTasty clinical trial, which enrolled both pediatric and adult patients who had achieved deep remission in the previously reported TASTI-MM and TASTI-E induction RCTs, demonstrating effectiveness for up to 24 weeks. Specifically, 86% participants maintained clinical remission, 86% exhibited radiological improvement assessed by the Intestinal Ultrasound with Segmental Activity Scoring, and endoscopic features, and 61% achieved complete endoscopic healing or presented only a few aphthous ulcers at ileocolonscopy or capsular endoscopy [45].

### 3.5. Ulcerative Colitis Exclusion Diet (UCED) and Other Food-Based Therapies in UC

While several food-based therapies have been proposed for CD, only a few studies have explored dietary interventions for the management of UC. Supporting a potential impact of diet on UC, Navas-Lòpez et al. explored dietary habits between pediatric patients reaching clinical remission and mucosal healing and those who did not. Interestingly, the authors described a superior dietary quality, characterized by a higher intake of unprocessed or minimally processed foods, fibers, and essential micronutrients, in children with clinical remission and mucosal healing. On contrast, patients without clinical remission and mucosal healing exhibited a higher consumption of ultra-processed foods and saturated fats [90]. Further supporting the adoption of a food-based therapeutic approaches for UC, harmful dietary components promote gut dysbiosis and may be consequently implied in UC pathogenesis. Such dietary interventions may aim to modulate gut microbiota composition and function, enhance intestinal barrier integrity, and influence innate immune responses.

Building upon this rationale, Sarbagili-Shabat et al. recently proposed the UCED, consisting of an initial 6–8-week phase that excludes red meat and processed foods, while emphasizing the inclusion of both mandatory foods, mainly fruits and vegetables, and permitted foods. The latter are either unrestricted, such as rice and potatoes, or allowed in defined quantities, such as chicken, eggs, yogurt, and pasta. The second phase of UCED is more permissive, allowing a broader variety of fruits and vegetables, together with the gradual reintroduction of grain products and certain legumes [46]. In a pilot trial involving 24 children with mild-to-moderate UC, the authors reported that the UCED was both effective and feasible for inducing remission. Specifically, the PUCAI decreased significantly at week 6 (35 (30–40) vs. 12.5 (5–20), *p* = 0.001), and clinical remission was achieved with UCED alone in nine of 24 participants (37.5%) [46].

Dietary approaches for UC have been poorly investigated also in adults. To date, only one additional food-based therapy for UC, termed the 4-strategies-to-SUlfide-Reduction (4-SURE) diet, has been proposed. This intervention is designed to modulate colonic fermentation and reduce the production of excess hydrogen sulfide, a metabolite with pro-inflammatory effect. Specifically, the diet is based on the following four central strategies: (1) to achieve an intake of 10–15 g/die of resistant starch and 5 g/die of slowly fermentable non-starch polysaccharide through the consumption of food sources rich in all forms of naturally occurring resistant starch, such as whole grains, and supplemental non-starch polysaccharide psyllium, in addition to fruits, vegetables, nuts, and legumes; (2) to limit the protein intake from animal and plant sources to 75–90 g/die; (3) to restrict the intake of sulfur-containing amino acids to ≤1.5–2.0 g/die; and (4) to avoid sulfite/sulfate, nitrite/nitrate, and carrageenan food additives. In a feasibility study involving 28 adults with mild-to-moderate UC, the 4-SURE diet demonstrated promising results: the intervention was well tolerated, with high adherence; clinical response was observed in 46% of patients; and endoscopic improvement occurred in 36% of participants. Moreover, fecal SCFA concentration increased by 69% (*p* < 0.001), accompanied by an improvement of QoL (*p* < 0.001) [91].

### 3.6. Plant-Based Diets (PBDs)

PBDs, ranging from semi-vegetarian to vegetarian and vegan patterns, have attracted increasing attention due to their potential anti-inflammatory properties and favorable modulation of the gut microbiota. These dietary models emphasize the intake of whole-plant foods rich in fiber, polyphenols, and antioxidants, while limiting or excluding animal products and processed foods [92].

Chiba et al. proposed a lacto-ovo-semi-vegetarian PBD, permitting fish once per week and meat once every 2 weeks, as an adjunct to infliximab in 35 adults and 11 pediatric patients with CD in a prospective single-arm study. Remission rates at 6 weeks were 96% and 100% by intention-to-treat and per-protocol analyses, respectively [47].

### 3.7. Low FODMAP Diet

The low FODMAP diet, commonly used for the management of irritable bowel syndrome, restricts low fermentable oligosaccharides, disaccharides, monosaccharides, and polyols. This dietary strategy has been only minimally investigated in patients with IBD.

Peng et al. conducted a systematic review including four RCTs evaluating the impact of a low FODMAP diet on maintaining remission in quiescent CD. Compared with a normal diet, the intervention showed no effect on stool consistency or inflammatory markers once remission was achieved, although it provided symptomatic relief of functional gastrointestinal symptoms and improved QoL [93].

### 3.8. IBD Anti-Inflammatory Diet (IBD-AID)

The IBD anti-inflammatory diet (IBD-AID) was proposed by Olendzki et al. in 2014 to reduce the frequency and severity of disease flares, and to promote obtaining and maintaining remission [94]. The IBD-AID is structured around five key principles: (1) restriction of certain carbohydrates, including lactose and refined or processed complex carbohydrates; (2) inclusion of prebiotics and probiotics; (3) distinction between saturated, trans, monounsaturated, and polyunsaturated fats; (4) individualized dietary assessment to identify nutrient deficiencies or intolerances; and (5) modification of food texture when necessary to enhance nutrient absorption and reduce intact fiber intake.

In a case series of 40 adults with IBD, this approach was associated with symptomatic improvement and medication reduction in 11 patients [94]. Another open-label study further supported the potential of the IBD-AID, demonstrating an enrichment of beneficial gut microbes, such as *Clostridia* and *Bacteroides*, typically depleted in IBD cohorts. However, the study did not analyze clinical outcomes [95].

### 3.9. Ketogenic Diets (KD)

There is growing interest in KD for IBD, as this dietary model may influence SCFA-producing bacterial populations. The classical KD is characterized by a strict restriction of carbohydrates and high fat intake, with fats providing approximately 90% of total energy. Evidence supporting KD use in IBD remains scarce, particularly in children [96].

To date, only a single case report has been published describing a paleolithic KD, consisting of animal fat, meat, offal, and eggs, with an approximate fat-to-protein ratio of 2:1. Specifically, the report detailed a 14-year-old boy with active, therapy-refractory CD who achieved clinical remission following implementation of the paleolithic KD, which was sustained even after azathioprine discontinuation [48].

### 3.10. Mediterranean Diet (MED)

The MED provides a rich source of bioactive compounds with potential immunomodulatory effect, such as a high intake of fresh fruits and vegetables, monounsaturated fats, complex carbohydrates, and lean proteins, while being low in ultra-processed foods, added sugar, and salt [97]. For these properties, MED has been associated with several favorable effects on gut health, such as an increased microbial diversity with relative abundance of SCFA-producing bacteria [98].

To date, only one RCT has evaluated the efficacy of MED in pediatric IBD. This study included 100 children and adolescents with mild-to-moderate CD or UC. After 12 weeks of adherence to MED, participants receiving this food-based therapy demonstrated significant reductions in clinical scores (PCDAI and Pediatric Ulcerative Colitis Activity Index-PUCAI), as well as in laboratory markers (CRP and FC), compared to those following a regular diet [49]. It is important to note, however, that the study did not report baseline concomitant medications, posing a potential confounding factor. Sigall Boneh et al. further explored the use of MED in children with CD in clinical remission under biological therapy. High adherence to MED, assessed by the KIDMED score, was significantly associated with lower FC levels (OR: 0.75; 95%CI: 0.6–0.95, *p* = 0.019), suggesting a beneficial role of this dietary pattern even in the maintenance phase of disease [50].

Additional evidence regarding the impact of MED in CD largely derives from studies in adult populations. Godny et al. investigated the effects of a modified version of the MED, termed the IBD-MED, in 271 adult patients with CD. The IBD-MED emphasizes fermented foods, potatoes, plant diversity, and a wide range of colors in fruits and color variety to promote microbial diversity and SCFA production. The study found that adherence to the IBD-MED was associated with a non-complicated disease course and significant improvements in disease activity; inflammatory markers such as CRP and FC; and gut microbiota composition, including an increase in SCFA-producing bacteria and a reduction in *Ruminoccocus gnavus* [99]. Furthermore, a longitudinal study involving adults with IBD, both CD and UC, showed that adherence to MED was associated with lower disease flares, reduced FC levels, and decreased corticosteroid use over a median follow-up of 27 months, although statistical significance was observed only among patients with UC [100]. Finally, Lewis et al. conducted a RCT comparing MED to SCD in adults with mild-to-moderate CD over 6 weeks. The study demonstrated that SCD was not superior to MED in achieving clinical remission and biochemical improvement, assessed by CRP and FC. Notably, although symptomatic remission was achieved by 46.5% patients receiving the SCD and by 43.5% receiving the MED (*p* = 0.77), none of the two diets allowed CRP response at week 6 or week 12, achieved by fewer than 11% of patients with both diets [101].

## 4. Pediatric-Specific Considerations on Food-Based Therapies

When considering food-based therapies in children and adolescents, several age-specific aspects deserve attention. While the primary goal of nutritional care in adults include the prevention of malnutrition, micronutrient deficiencies, and osteoporosis, nutritional management in pediatric patients must also support optimal growth and development [102]. However, evidence regarding nutritional adequacy of food-based therapies in pediatric IBD remains limited. Therefore, given the restrictive nature of some dietary approaches, a careful monitoring of nutritional status, growth parameters, and dietary intake should be warranted, especially in children at increased risk of malnutrition. Parallelly, education of pediatric patients and their families represents a key component of successful dietary management. Several practical tools may facilitate adherence and ensure nutritional adequacy, including recipes and videos—mainly available for the CDED; shopping guides; dedicated support lines; and smartphone apps that allow dietary intake to be recorded and subsequently reviewed by dietitians and clinicians [103].

Furthermore, pediatric age encompasses a broad developmental spectrum, ranging from young children to adolescents, and age-related differences may substantially influence dietary habits and adherence to dietary interventions. In adolescents, adherence to food-based therapies may be challenged by a variety of psychological, social, and dietary-restriction-related reasons. Notably, a high proportion, reaching nearly 60% of adolescents with IBD, exhibit dietary modifications, most commonly food avoidance, which may adversely affect psychological well-being and acceptance of changes in the dietary regimen [104,105]. Moreover, also younger children may experience both maladaptive eating behaviors, such as “picky eating”, and eating disorders, such as the avoidant restrictive food intake disorder (ARFID), both of which may negatively affect adherence to prescribed food-based therapies [103]. Accordingly, multidisciplinary management should be considered, including access to psychologist with experience in pediatric IBD, when appropriate [106]. Psychological support may be particularly valuable for patients experiencing eating disorders, maladaptive eating behaviors, or disease-related psychological distress.

## 5. Discussion

Food-based therapies represent a promising approach in the management of IBD, particularly CD, as they may overcome the poor tolerance associated to EEN while aiming to induce, and in some cases even maintain, clinical remission. Based on the hypothesis that disease pathogenesis is at least partially driven by exposure to harmful dietary components, such as the ultra-processed foods, several dietary strategies have been proposed to modulate gut microbiota function and composition, thereby ameliorating intestinal inflammation. Although initial results are encouraging, evidence supporting most dietary regimens remains limited. Features of the dietary regimens discussed in this narrative review are summarized in Table 2.

Among food-based therapies, the CDED with PED is currently the most extensively studied in pediatric CD, showing promising results. Several studies have demonstrated both safety and efficacy of this intervention as a first-line therapy in mild-to-moderate CD, and as a rescue therapy in those with refractory disease. In addition to its therapeutic benefits, CDED with PEN tends to be better accepted by children and adolescents than EEN. Nevertheless, adherence issues may arise, particularly during phase 1, which involves a stricter dietary regimen. Thus, comprehensive education and support for both patients and their families are essential to ensure compliance.

The MED also represents an appealing dietary strategy, as its less restrictive nature compared with other food-based therapies may improve acceptance and tolerability among pediatric patients. However, as previously noted, evidence supporting the use of MED in pediatric IBD remains limited, and data extrapolated from adult studies still leave uncertainties regarding its true effectiveness in inducing remission. Nonetheless, the numerous advantages of the MED, including its balanced nutritional profile, broader food variety, and strong cultural acceptability, make it is a reasonable dietary recommendation for children and adolescents with IBD, at least as an adjunct to conventional therapies. In support of this approach, the recent clinical practice update from the American Gastroenterological Association endorses the MED as a suitable, long-term dietary pattern for IBD patients [97]. Notably, an Italian case–control study revealed that children with IBD often exhibit suboptimal dietary habits, characterized by inadequate energy and nutrient intake, and poor adherence to a high-quality Mediterranean pattern [107]. Therefore, when prescribing the MED, it is advisable to provide nutritional counselling to promote healthy lifestyle behaviors consistent with dietary model, regardless of concomitant medical therapy.

Similarly, the T&H diet represents another promising whole-food approach in children with CD. Preliminary findings suggest potential benefits in pediatric CD management, along with good tolerability. However, current evidence remains scarce, and further studies are required to better assess its efficacy. As with the MED, the T&H diet may serve as an adjunctive therapy alongside pharmacological treatments.

**Figure 1:**
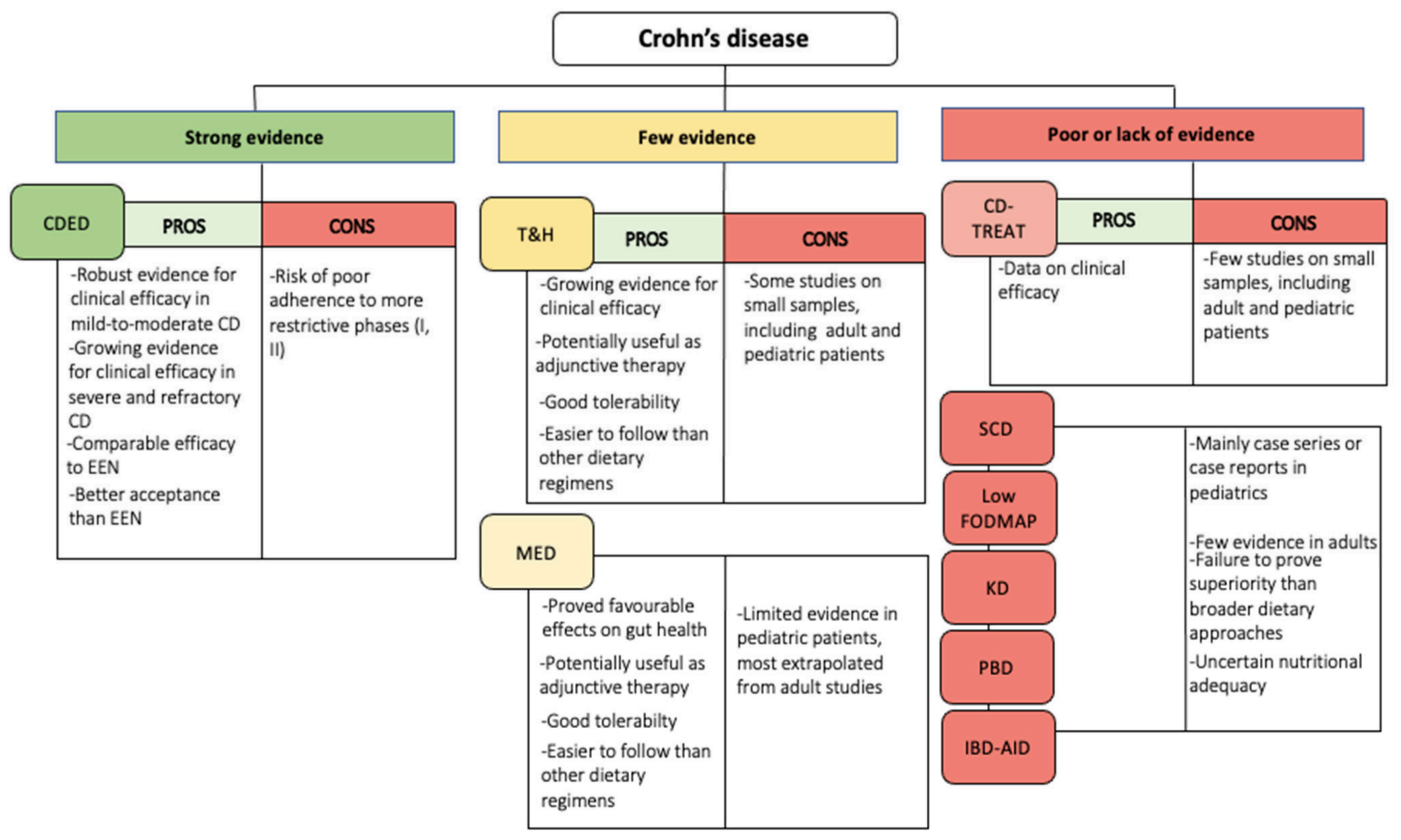
Suggestions on food-based therapies for Crohn’s disease based on current evidence. Abbreviations: CDED, Crohn’s Disease Exclusion Diet; CD, Crohn’s disease; EEN, exclusive enteral nutrition; T&H, Tasty and Healthy Diet; MED, Mediterranean diet; CD-TREAT, Crohn’s disease treatment-with-EATing; SCD, specific carbohydrate diet; KD, ketogenic diet; PBD, plant-based diet; IBD-AID, IBD anti-inflammatory diet.

Additional food-based dietary interventions for IBD include the SCD, KD, PBD, and IBD-AID. However, evidence supporting these approaches is limited, largely derived from studies with small sample sizes and primarily adult populations. Importantly, their implementation in clinical practice may not yield favorable outcomes in pediatric IBD. For example, the current literature failed to demonstrate the superiority of the SCD over the MED, which provides a whole-food dietary pattern that is broader and more acceptable to children and adolescents, and consequently may be associated with better tolerance [101]. Furthermore, the limited available data not only fail to support the efficacy of these dietary strategies, but also do not establish their nutritional adequacy. As a consequence, potential nutrient deficiencies cannot be excluded. In conclusion, we think that it is reasonable to consider broader whole-food options, such as the MED or the T&H diet, as a preferable option, particularly as an adjunct to other therapeutic approaches, even if their capacity to induce clinical remission remains unproven. Figure 1 summarized our suggestions in the choice of food-based therapies for CD, based on the current literature.

With respect to UC, the UCED has emerged as a promising nutritional strategy that may complement conventional and biologic therapies. Nevertheless, as with most food-based therapies, current evidence remains limited, and further investigation is warranted before UCED can be recommended for routine clinical practice.

Notably, consideration should be given to adherence to dietary treatment, which may be compromised, foremost by poor palatability or limited acceptability of the proposed foods. Moreover, economic and cultural factors should also be taken into account. In particular, adopting a healthy dietary pattern, including the reduction or elimination of ultra-processed foods, which are cheaper and more available than healthier ingredients, often requires financial resources that may not be readily available to all families. In addition, individuals from certain ethnic backgrounds may experience difficulties adhering to specific nutritional strategies, such as the more restrictive phases of the CDED, in which the number of mandatory foods is particularly limited. Consequently, adapting the CDED to different cultural and ethnic backgrounds is essential to improve patient adherence and maximize its effectiveness. In this context, structured training programs for healthcare providers may further enhance the accuracy and consistency of dietary therapy implementation [103,106].

## 6. Conclusions

There is currently growing interest in nutritional therapies for pediatric IBD, as these strategies, when effective, may promote mucosal healing while reducing children’s exposure to pharmacological treatments that are not devoid of adverse effects. Several approaches have been proposed, all grounded in the concept of modulating intestinal inflammation primarily through the regulation of the gut microbiota. This objective is pursued in each dietary intervention by applying two fundamental principles: the introduction of foods that may exert beneficial effects on gastrointestinal homeostasis and the exclusion of potentially harmful dietary components.

The role of diet in CD has been extensively investigated, whereas only few dietary therapeutic approaches for UC have been proposed. While CDED is the most studied food-based therapy for CD, some whole-food strategies such as the T&H diet and the MED have been proposed. The latter, if confirmed to be effective as adjunctive therapies in future studies, could represent a valuable option for children and adolescents, as they are less restrictive and therefore potentially more acceptable. In contrast, evidence supporting other dietary regimens remains very limited, rendering them unsuitable for routine clinical use and restricting their application to experimental contexts.

Similarly, evidence regarding food-based therapy in UC remains scarce. Nonetheless, further research is warranted, as children with UC may experience frequent flares requiring corticosteroid therapy. Whether the optimization of UC management through complementary nutritional strategies may provide some benefits to pediatric populations could represent an interesting field for future research.

In conclusion, food-based strategies represent a promising therapeutic avenue in pediatric IBD, currently with stronger evidence in CD but with increasing attention also in UC interventions, such as the UCED.

Nevertheless, nutritional management is becoming increasingly relevant in pediatric IBD clinical practice, and it should be further emphasized, both through dietary approaches aimed at inducing and maintaining disease remission potentially as monotherapy, as in the case of the CDED for CD, and through the promotion of dietary habits with potential anti-inflammatory properties, such as the MED. In this context, the identification of an appropriate, safe, and effective nutritional therapy should represent an integral part of a multidisciplinary approach, involving both gastroenterologists and dietitians, pointing to a personalized management of each IBD patient, with the aim of improving disease control, nutritional status, growth, and long-term outcomes.

## 7. Future Perspectives

Over the past decade, interest in food-based therapies for IBD has grown substantially. Dietary therapies are particularly appealing in such conditions because, besides their nutritional impact, they have demonstrated the ability to induce remission essentially without any major side effects. Within the expanding armamentarium of dietary therapies, however, major unanswered questions remain: how do we identify patients who are more likely to benefit from a certain regimen, and how can we maximize adherence to sustain the achieved remission? The considerable variability in treatment response, which is intrinsically related to the complex nature of IBD, makes the identification of dietary responders particularly challenging. One of the possible factors to consider, and upon which most of the evidence has focused, is the gut microbiome [20]. Recent data suggest that specific baseline microbial and functional signatures—such as lower alpha diversity and higher abundances of taxa like *Alistipes* and *Faecalibacterium*—might eventually help identify which patients are most likely to achieve sustained clinical remission on exclusion diets. Furthermore, successful dietary intervention appears to shift these initial dysbiotic profiles toward a much healthier, anti-inflammatory state [108].

Alongside the microbiome, another possible factor to consider involves the metabolome. Recent analyses of pediatric cohorts reveal that successful dietary interventions trigger functional shifts toward a healthy metabotype, even though the early clinical response appears largely independent of short-chain fatty acid or bile acid concentrations [20,109,110]. Specifically, sustained remission correlates with persistent reductions in pro-inflammatory kynurenine pathway metabolites and succinate synthesis components, alongside increases in serotonin pathway metabolites like melatonin. Consequently, distinct metabolite ratios—such as the kynurenine-to-melatonin ratio—demonstrate high discriminatory power and could eventually serve as prognostic biomarkers to identify true dietary responders. Nevertheless, similar to microbial signatures, these metabolomic observations stem from preliminary, targeted analyses and demand extensive validation in larger, longitudinal cohorts before they can be translated into routine practice.

Ultimately, integrating these diverse layers of biological data could offer a much deeper understanding of the complex mechanisms guiding dietary treatments in IBD. For example, a recent proof-of-concept study demonstrated that a machine-learning model combining multi-omics data—including the fecal microbiome, serum metabolomics, and lipidomics—could successfully predict pediatric patient responses to exclusive enteral nutrition with high accuracy. While this research is still in its early stages and requires further validation, such integrated multi-omics models highlight the highly promising future of precision nutrition and personalized care in managing Crohn’s disease [111].

## Figures and Tables

**Table 1:** Studies on food-based therapies in children with inflammatory bowel diseases.

First Author (Year)	Study Design	Population (I: Intervention; C: Control)	Intervention (I: Intervention; C: Control)	Key Findings
**CDED**				
Levine et al., 2019 [19]	RCT	- I: 40 pediatric patients with CD - C: 38 pediatric patients with CD	- I: CDED + PEN (12 weeks) - C: EEN (12 weeks)	- 75.6% corticosteroid-free remission with CDED + PEN vs. 45.1% with EEN - 97.5% tolerance with CDED + PEN vs. 73.6% with EEN
Verburgt et al., 2023 [20]	RCT	- I: 23 pediatric patients with CD - I: 18 pediatric patients with CD	- I: CDED + PEN (12 weeks) - I: EEN (12 weeks)	- Improvement of gut microbiota at 12-weeks, particularly with reduced Proteobacteria, in both groups
Rohani et al., 2025 [21]	RCT	- I: 30 pediatric patients with CD - C: 30 pediatric patients with CD	- I: CDED + PEN (8 weeks) - C: CDED alone (8 weeks)	- Higher increase in *Bifidobacterium* and *Lactobacillus* with CDED + PEN vs. CDED alone - Greater decrease in *E. coli* and *Fusobacterium* with CDED + PEN vs. CDED alone
Sigall-Boneh et al., 2014 [22]	Retrospective study	34 children and 13 young adults with CD	CDED + PEN (12 weeks)	- 78.7% clinical response - 70.2% clinical remission (70.0% in children) - 70.0% normalization of FC
Niseteo et al., 2022 [23]	Retrospective study	- I: 20 pediatric CD patients - C: 41 pediatric CD patients	- I: CDED + PEN (6–8 weeks) - C: EEN (6 weeks)	- 75.0% clinical remission with CDED + PEN vs. 68.9% with EEN - Greater weight gain and increase in BMI z-score with CDED + PEN vs. EEN
Sigall Boneh et al., 2025 [24]	RCT	- I: 30 pediatric patients with CD - C: 26 pediatric patients with CD	- I: PEN 2 weeks followed by CDED + PEN - C: EEN 8 weeks followed by PEN + free diet	- 70% corticosteroid-free remission with CDED + PEN vs. 61.5% with EEN (week 14) - 60% clinical remission with CDED + PEN vs. 42% with EEN (week 24) - Greater increase in BMI z-score with CDED + PEN vs. EEN
Sigall Boneh et al., 2017 [25]	Retrospective study	11 children and 10 young adults with refractory CD	- 12 patients receiving CDED + PEN (12 weeks) - 4 patients receiving CDED (12 weeks) - 5 patients receiving EEN followed by CDED (2 + 12 weeks)	- 61.9% clinical remission - 52.9% clinical remission in patients failing double biologic therapy - Improvement in CRP and serum albumin
Scarallo et al., 2021 [26]	Case series	5 children with CD (2 with refractory CD)	CDED + PEN (various duration)	CDED + PEN as a safe and effective therapeutic option as induction and maintenance therapy
Scarallo et al., 2024 [27]	Retrospective study	66 pediatric patients with CD	- 60 patients receiving CDED + PEN as monotherapy (8 weeks) - 26 patients receiving CDED + PEN as add-on therapy (8 weeks)	- 69.7% clinical remission - 66.7% CRP normalization - 27.2% FC normalization - Lower clinical response rates with EIM
Jijón Andrade et al., 2023 [28]	Retrospective study	15 pediatric patients with CD	CDED + PEN (24 weeks)	- 87% clinical remission in treatment-naïve-patients - 60.0% clinical remission in patients with refractory CD - Improvement in erythrocyte sedimentation rate and hemoglobin levels
Pochesci et al., 2025 [29]	Retrospective study	- I: 16 pediatric patients with CD - C: 21 pediatric patients with CD	- I: CDED + PEN + ATA (52 weeks) - C: ATA (52 weeks)	- Greater improvement in CRP and ESR in CDED + PEN + ATA vs. ATA (8 weeks) - Greater improvement in weighted PCDAI in CDED + PEN + ATA vs. ATA (8 weeks) - Greater improvement in weight in CDED + PEN + ATA vs. ATA (52 weeks)
Urlep et al., 2023 [30]	Prospective study	- I: 14 pediatric patients with CD - C: 19 pediatric patients with CD	- I: CDED + PEN (6 weeks) - C: EEN (6 weeks)	- 84.6% endoscopic response with CDED + PEN vs. 68.8% with EEN - 38.5% mucosal healing with CDED + PEN vs. 43.8% with EEN
Martín-Masot et al., 2023 [31]	Prospective study	24 pediatric patients with CD	CDED (52 weeks)	- Improvement of the MINI index - Improvement in the dietary pattern with reduced consumption of ultra-processed foods
Lev-Tzion et al., 2021 [32]	RCT	- I: 23 pediatric patients with CD - C: 22 pediatric patients with CD	- I: CDED + PEN (24 weeks) - C: EEN 6 weeks followed by PEN + free diet (24 weeks)	- Improvement of C-Propeptide of Type I Procollagen levels in both groups
Wijers et al., 2025 [33]	Retrospective study	52 children and 17 adults with CD	CDED + PEN	- Good experience with CDED + PEN (83% patients that may recommend CDED + PEN to others; 63% patients that may reconsider starting CDED + PEN again)
Alsarhan et al., 2023 [34]	Retrospective study	32 patients with CD	CDED + PEN	57% of patient continued CDED beyond 12 weeks
**SCD**				
Suskind et al., 2014 [35]	Case series	7 children with CD	SCD (5–30 months)	Improvement in serum albumin, CRP, hematocrit, and FC
Obih et al., 2016 [36]	Retrospective study	26 pediatric patients with IBD (20 CD; 6 UC)	SCD (3–48 months)	Reduction of PCDAI and PUCAI over a 6-months period
Wahbeh et al., 2017 [37]	Case series	7 children with CD	SCD or modified SCD (median duration 26 months)	- No clinical activity and normalization of CRP in all patients - No cases of mucosal healing
Britto et al., 2020 [38]	Case series	3 children with CD	SCD (52 weeks)	- Clinical remission and normalization of CF - No cases of mucosal healing
Cohen et al., 2014 [39]	Case series	9 children with CD	SCD (12 weeks)	- Clinical improvement (reduction of PCDAI) - Mucosal improvement (reduction of Lewis score)
Simon et al., 2023 [40]	Case report	1 child with CD	SCD (52 weeks)	Clinical, biochemical (FC, CRP, serum albumin, erythrocyte sedimentation rate, and hemoglobin), endoscopic, and histologic remission
**CD-TREAT**				
Svolos et al., 2019 [41]	Prospective study	5 pediatric patients with CD	CD-TREAT (8 weeks)	- 80.0% clinical response - 60.0% clinical remission - FC reduction
Svolos et al., 2022 [42]	Prospective study	25 pediatric patients and 32 adults with CD	CD-TREAT (8 weeks)	- 78% clinical remission - 85% clinical remission - Improvement in QoL -FC reduction
**T&H Dietary Approach**				
Aharoni-Frutkoff et al., 2025 [43]	RCT	83 biologic-naïve pediatric and adult patients with CD (6–25 years): - I: 41 patients - C: 42 patients	-I: T&H (8 weeks) -C: EEN (8 weeks)	- 56.0% clinical remission with T&H vs. 38.0% with EEN - 88.0% tolerance with T&H vs. 52.0% with EEN - Improvement of the gut microbiota with T&H
Plotkin et al., 2025 [44]	RCT	46 pediatric and adult patients with CD (6–40 years)	I: T&H (16 weeks) C: habitual diet 8 weeks, followed by T&H 8 weeks	- 37% FC response with T&H vs. 15% with habitual diet at 8 weeks - Greater CRP reduction with T&H vs. habitual diet at 8 weeks - Greater MINI index reduction with T&H vs. habitual diet at 8 weeks - In habit group switching to T&H, higher rates of calprotectin < 250 mcg/g at week 16 compared to week 8 (47% vs. 17%) - In habit group switching to T&H, higher rates of MINI index < 8 at week 16 compared to week 8 (60% vs. 20%, *p* = 0.0013).
Plotkin et al., 2026 [45]	Clinical trial	10 adults and 33 children with CD	T&H (24 weeks)	- 86% clinical remission - 86% radiological improvement - 61% endoscopic healing or few aphthous ulcers
**UCED**				
Sarbagili-Shabat et al., 2021 [46]	Prospective study	23 children with UC	UCED (6 weeks)	- 37.5% clinical remission - FC reduction
**PBD**				
Chiba et al., 2017 [47]	Prospective study	35 adults and 11 children with CD	PBD + Infliximab (6 weeks)	96.0–100.0% clinical remission (intention-to-treat and per-protocol analyses)
**KD**				
Csiba-Tóth et al., 2016 [48]	Case report	1 adolescent with active, refractory CD	KD + Azathioprine (discontinued within 2 weeks) (15 months)	- Clinical remission
**MED**				
El Amrousy et al., 2022 [49]	RCT	- I: 26 pediatric patients with CD and 24 pediatric patients with UC - C: 28 pediatric patients with CD and 22 pediatric patients with UC	- I: MED (12 weeks) * - C: regular diet (12 weeks) *	- 28.0% clinical remission with MED vs. 16.0% with regular diet - Greater decrease in PCDAI and PUCAI with MED vs. regular diet
Sigall Boneh et al., 2024 [50]	Cross-sectional study	99 pediatric patients with CD (in clinical remission under biological therapy)	MED + biologic therapy	- Association between adherence to MED and FC reduction - Inversely association between vegetable consumption and FC levels

* The study did not report baseline concomitant therapies. Abbreviations: CDED, Crohn’s Disease Exclusion Diet; PEN, partial enteral nutrition; CD, Crohn’s disease; EEN, exclusive enteral nutrition; RCT, randomized controlled trial; FC, fecal calprotectin; CRP, C-reactive protein; ATA, anti-TNFa agent; ESR, erythrocyte sedimentation rate; PCDAI, Pediatric Crohn’s Disease Activity Index; SCD, specific carbohydrate diet; UC, ulcerative colitis; PUCAI, Pediatric Ulcerative Colitis Activity Index; CD-TREAT, Crohn’s disease treatment-with-EATing; QoL, quality of life; T&H, Tasty and Healthy; UCED, Ulcerative Colitis Exclusion Diet; PBD, plant-based diet; KD, ketogenic diet; MED, Mediterranean diet.

**Table 2:** Features of food-based therapies in inflammatory bowel diseases.

	Include	Avoid	Polymeric Formula
**CDED**	- Phase I: 5 mandatory foods (chicken, eggs, potatoes, bananas, apples), permitted ingredients with defined quantities (fish, rice, avocado, strawberries, melon, tomatoes, cucumbers, carrots, spinaches, olive oil, certain herbs, seasonings, and sweeteners) - Phase II: broader variability of fibers, controlled reintroduction of gluten and red meat - Phase III: whole-food diet emphasizing fruit and vegetables intake	Excluded in all phases: processed foods, saturated fatty acids, refined sugars, emulsifiers (e.g., carrageenan, maltodextrin, sulphites)	Included
**T&H**	Fresh, unprocessed foods (e.g., Fruits and vegetables, legumes, rice, gluten-free grais, eggs, poultry, fish)	Processed foods, animal fats, gluten	Not included
**MED**	Fruit and vegetables, whole grains, monousatured fats (e.g., olive oil), moderate amounts of poultry, fish, and dairy products	Processed foods, high intake of red meat, refined sugars, high intake of salt	Not included
**CD-TREAT**	Macronutrients, vitamins, minerals, and fiber combined for recreating EEN composition	Dairy products, gluten, alcohol	Not included
**SCD**		Sweeteners (except for honey) and candies, all grains and grain-based products (including rice and oats), most legumes, potatoes, processed meat, processed vegetables, dairy products (except for hard cheeses and yogurt fermented more than 24 h), processed foods	Not included
**Low FODMAP diet**		Fermentable foods, specifically oligosaccharides, disaccharides, monosachharides, polyols	Not included
**IBD-AID**		Trans fats, emulsifiers (e.g., carrageenan, maltodextrin, sulphites), refined sugars, processed food, milk and fresh cheeses	Not included
**PBD**	Semi-vegetarian to vegetarian and vegan pattern, emphasizing the intake of whole-plant foods rich in fibres, polyphenols, antioxidants	Processed foods, animal products (limited or excluded)	Not included
**KD**	High fat intake (90% of total energy)	Carbohydrates (strict restriction)	Not included
**UCED**	- Phase I: mandatory foods (some fruits and vegetables), unrestricted permitted foods (rice, potatoes), allowed foods, with defined quantities (chicken, eggs, yogurt, pasta) - Phase II: broader variability of fruits and vegetables, grain products, certain legumes	Processed foods, red meat	Not included
**4-SURE**	Resistant starch and slowly fermentable non-starch polysaccharide (e.g., whole grains, supplemental non-starch polysaccharide psyllium, fruits, vegetables, nuts, and legumes)	High-protein intake, high sulphur-containing amino acids intake, emulsifiers (e.g., sulphite, carrageenan)	Not included

Abbreviations: CDED, Crohn’s Disease Exclusion Diet; T&H, Tasty and Healthy diet; MED, Mediterranean diet; CD-TREAT, Crohn’s disease treatment-with-EATing; SCD, specific carbohydrate diet; IBD-AID, IBD anti-inflammatory diet; PBD, plant-based diet; KD, ketogenic diet; UCED, Ulcerative Colitis Exclusion Diet; 4-SURE, 4-strategies-to-SUlfide-Reduction Diet.

## Data Availability

No new data were created or analyzed in this study. Data sharing is not applicable to this article.

## Abbreviations

The following abbreviations are used in this manuscript:

IBD	Inflammatory bowel disease
UC	Ulcerative colitis
CD	Crohn’s disease
EEN	Exclusive enteral nutrition
RCT	Randomized controlled trial
SCFAs	Short-chain fatty acids
WD	Western diet
OR	Odds ratio
CI	Confidence interval
HR	Hazard ratio
CRP	C-reactive protein
FC	Fecal calprotectin
PUFAs	Polyunsaturated fatty acids
CDED	Crohn’s disease exclusion diet
PEN	Partial enteral nutrition
PCDAI	Pediatric Crohn’s disease activity index
SCD	Specific carbohydrate diet
mSCD	Modified specific carbohydrate diet
MED	Mediterranean diet
PUCAI	Pediatric Ulcerative Colitis Activity Index
CD-TREAT	Crohn’s disease treatment-with-EATing
QoL	Quality of life
CDAI	Crohn’s Disease Activity Index

## References

[1] Ghione S., Sarter H., Fumery M., Armengol-Debeir L., Savoye G., Ley D., Spyckerelle C., Pariente B., Peyrin-Biroulet L., Turck D. (2018). Dramatic Increase in Incidence of Ulcerative Colitis and Crohn’s Disease (1988–2011): A Population-Based Study of French Adolescents. Am. J. Gastroenterol..

[2] Benchimol E.I., Manuel D.G., Guttmann A., Nguyen G.C., Mojaverian N., Quach P., Mack D.R. (2014). Changing age demographics of inflammatory bowel disease in Ontario, Canada: A population-based cohort study of epidemiology trends. Inflamm. Bowel Dis..

[3] Scarallo L., Griffiths A.M. (2022). Medical therapy of paediatric inflammatory bowel disease. Nat. Rev. Gastroenterol. Hepatol..

[4] Alvisi P., Labriola F., Scarallo L., Gandullia P., Knafelz D., Bramuzzo M., Zuin G., Pastore M.R., Illiceto M.T., Miele E. (2022). Epidemiological trends of pediatric IBD in Italy: A 10-year analysis of the Italian society of pediatric gastroenterology, hepatology and nutrition registry. Dig. Liver Dis..

[5] Huang H., Fang M., Jostins L., Mirkov M.U., Boucher G., Anderson C.A., Andersen V., Cleynen I., Cortes A., Crins F. (2017). Fine-mapping inflammatory bowel disease loci to single-variant resolution. Nature.

[6] Peters L.A., Perrigoue J., Mortha A., Iuga A., Song W.-M., Neiman E.M., Llewellyn S.R., Di Narzo A., Kidd B.A., Telesco S.E. (2017). A functional genomics predictive network model identifies regulators of inflammatory bowel disease. Nat. Genet..

[7] Sýkora J., Pomahačová R., Kreslová M., Cvalínová D., Štych P., Schwarz J. (2018). Current global trends in the incidence of pediatric-onset inflammatory bowel disease. World J. Gastroenterol..

[8] Kuenzig M.E., Fung S.G., Marderfeld L., Mak J.W., Kaplan G.G., Ng S.C., Wilson D.C., Cameron F., Henderson P., Kotze P.G. (2022). Twenty-first Century Trends in the Global Epidemiology of Pediatric-Onset Inflammatory Bowel Disease: Systematic Review. Gastroenterology.

[9] Loftus E.V. (2004). Clinical epidemiology of inflammatory bowel disease: Incidence, prevalence, and environmental influences. Gastroenterology.

[10] Khan R., Kuenzig M.E., Benchimol E.I. (2023). Epidemiology of Pediatric Inflammatory Bowel Disease. Gastroenterol. Clin. N. Am..

[11] Hou J.K., Abraham B., El-Serag H. (2011). Dietary intake and risk of developing inflammatory bowel disease: A systematic review of the literature. Am. J. Gastroenterol..

[12] Levine A., Boneh R.S., Wine E. (2018). Evolving role of diet in the pathogenesis and treatment of inflammatory bowel diseases. Gut.

[13] Ko Y., Butcher R., Leong R.W. (2014). Epidemiological studies of migration and environmental risk factors in the inflammatory bowel diseases. World J. Gastroenterol..

[14] Agrawal M., Burisch J., Colombel J.-F., Shah S.C. (2020). Viewpoint: Inflammatory Bowel Diseases Among Immigrants from Low- to High-Incidence Countries: Opportunities and Considerations. J. Crohn’s Colitis.

[15] Van Rheenen P.F., Aloi M., Assa A., Bronsky J., Escher J.C., Fagerberg U.L., Gasparetto M., Gerasimidis K., Griffiths A., Henderson P. (2021). The Medical Management of Paediatric Crohn’s Disease: An ECCO-ESPGHAN Guideline Update. J. Crohn’s Colitis.

[16] Turner D., Ruemmele F.M., Orlanski-Meyer E., Griffiths A.M., de Carpi J.M., Bronsky J., Veres G., Aloi M., Strisciuglio C., Braegger C.P. (2018). Management of paediatric ulcerative colitis, part 1: Ambulatory Care-An Evidence-Based Guideline from European Crohn’s and Colitis Organization and European Society of Paediatric Gastroenterology, Hepatology and Nutrition. J. Pediatr. Gastroenterol. Nutr..

[17] Swaminath A., Feathers A., Ananthakrishnan A.N., Falzon L., Ferry S.L. (2017). Systematic review with meta-analysis: Enteral nutrition therapy for the induction of remission in paediatric Crohn’s disease. Aliment. Pharmacol. Ther..

[18] Borrelli O., Cordischi L., Cirulli M., Paganelli M., Labalestra V., Uccini S., Russo P.M., Cucchiara S. (2006). Polymeric diet alone versus corticosteroids in the treatment of active pediatric Crohn’s disease: A randomized controlled open-label Trial. Clin. Gastroenterol. Hepatol..

[19] Levine A., Wine E., Assa A., Boneh R.S., Shaoul R., Kori M., Cohen S., Peleg S., Shamaly H., On A. (2019). Crohn’s Disease Exclusion Diet Plus Partial Enteral Nutrition Induces Sustained Remission in a Randomized Controlled Trial. Gastroenterology.

[20] Verburgt C.M., Dunn K.A., Ghiboub M., Lewis J.D., Wine E., Boneh R.S., Gerasimidis K., Shamir R., Penny S., Pinto D.M. (2023). Successful Dietary Therapy in Paediatric Crohn’s Disease is Associated with Shifts in Bacterial Dysbiosis and Inflammatory Metabotype Towards Healthy Controls. J. Crohn’s Colitis.

[21] Rohani P., Ansari A., Shaygantabar M., Hekmatdoost A., Sohouli M.H. (2025). The Effects of the Crohn’s Disease Exclusion Diet (CDED) Alone Versus CDED Plus Partial Enteral Nutrition (PEN) on Gut Microbiome Composition in Pediatric CD Patients. Microbiologyopen.

[22] Sigall-Boneh R., Pfeffer-Gik T., Segal I., Zangen T., Boaz M., Levine A. (2014). Partial enteral nutrition with a Crohnʼs disease exclusion diet is effective for induction of remission in children and young adults with Crohn’s disease. Inflamm. Bowel Dis..

[23] Niseteo T., Sila S., Trivić I., Mišak Z., Kolaček S., Hojsak I. (2022). Modified Crohn’s disease exclusion diet is equally effective as exclusive enteral nutrition: Real-world data. Nutr. Clin. Pr..

[24] Sigall Boneh R., Navas-López V.M., Hussey S., Pujol-Muncunill G., Lawrence S., Rolandsdotter H., Otley A., Martín-De-Carpi J., Abramas L., Herrador-López M. (2025). Modified Crohn’s Disease Exclusion Diet Maintains Remission in Pediatric Crohn’s Disease: Randomized Controlled Trial. Clin. Gastroenterol. Hepatol..

[25] Sigall Boneh R., Shabat C.S., Yanai H., Chermesh I., Ben Avraham S., Boaz M., Levine A. (2017). Dietary Therapy with the Crohn’s Disease Exclusion Diet is a Successful Strategy for Induction of Remission in Children and Adults Failing Biological Therapy. J. Crohn’s Colitis.

[26] Scarallo L., Banci E., Pierattini V., Lionetti P. (2021). Crohn’s disease exclusion diet in children with Crohn’s disease: A case series. Curr. Med. Res. Opin..

[27] Scarallo L., Banci E., De Blasi A., Paci M., Renzo S., Naldini S., Barp J., Pochesci S., Fioretti L., Pasquini B. (2024). A real-life pediatric experience of Crohn’s disease exclusion diet at disease onset and in refractory patients. J. Pediatr. Gastroenterol. Nutr..

[28] Jijón Andrade M.C., Muncunill G.P., Ruf A.L., Carnero L.Á., Miravet V.V., Arenas D.G., Castillo N.E., de Carpi J.M. (2023). Efficacy of Crohn’s disease exclusion diet in treatment -naïve children and children progressed on biological therapy: A retrospective chart review. BMC Gastroenterol..

[29] Pochesci S., Scarallo L., Miraglia T., Lionetti P. (2025). Crohn’s disease exclusion diet as an add-on to antitumor necrosis factor-α therapy in children with moderate-to-severe Crohn’s disease. J. Pediatr. Gastroenterol. Nutr..

[30] Urlep D., Orel R., Kunstek P., Benedik E. (2023). Treatment of Active Crohn’s Disease in Children Using Partial Enteral Nutrition Combined with a Modified Crohn’s Disease Exclusion Diet: A Pilot Prospective Cohort Trial on Clinical and Endoscopic Outcomes. Nutrients.

[31] Martín-Masot R., Herrador-López M., Navas-López V.M. (2023). Dietary Habit Modifications in Paediatric Patients After One Year of Treatment with the Crohn’s Disease Exclusion Diet. Nutrients.

[32] Lev-Tzion R., Ben-Moshe T., Abitbol G., Ledder O., Peleg S., Millman P., Shaoul R., Kori M., Assa A., Cohen S. (2021). The Effect of Nutritional Therapy on Bone Mineral Density and Bone Metabolism in Pediatric Crohn Disease. J. Pediatr. Gastroenterol. Nutr..

[33] Wijers F.T.R., van Zundert S.M.C., Verburgt C.M., van der Kruk N., Van Limbergen J.E., Wierdsma N.J. (2025). Patient experiences with and adherence to Crohn’s disease exclusion diet in Dutch Crohn’s disease patients: A cohort study. Ther. Adv. Gastroenterol..

[34] Alsarhan A., Aljasmi R., Ajaka N., Krishnamurthy B., Kader A., Aljasmi M., Nahdi N., Malik E., Murbati B., Aljabri E. (2023). Challenges in Managing Paediatric Crohn’s Disease with Crohn’s Disease Exclusion Diet (CDED): The First Single-Center Study in the United Arab Emirates. Cureus.

[35] Suskind D.L., Wahbeh G., Gregory N., Vendettuoli H., Christie D. (2014). Nutritional therapy in pediatric Crohn disease: The specific carbohydrate diet. J. Pediatr. Gastroenterol. Nutr..

[36] Obih C., Wahbeh G., Lee D., Braly K., Giefer M., Shaffer M.L., Nielson H., Suskind D.L. (2016). Specific carbohydrate diet for pediatric inflammatory bowel disease in clinical practice within an academic IBD center. Nutrition.

[37] Wahbeh G.T., Ward B.T., Lee D.Y., Giefer M.J., Suskind D.L. (2017). Lack of Mucosal Healing From Modified Specific Carbohydrate Diet in Pediatric Patients with Crohn Disease. J. Pediatr. Gastroenterol. Nutr..

[38] Britto S.L., Kellermayer R. (2020). Durable Clinical and Biochemical but Not Endoscopic Remission in Pediatric Crohn’s Disease on Specific Carbohydrate Diet Monotherapy. Ann. Clin. Lab. Sci..

[39] Cohen S.A., Gold B.D., Oliva S., Lewis J., Stallworth A., Koch B., Eshee L., Mason D. (2014). Clinical and mucosal improvement with specific carbohydrate diet in pediatric Crohn disease. J. Pediatr. Gastroenterol. Nutr..

[40] Simon D., Patel K., Masand P., Kellermayer R. (2023). Food for Thought: Remission of Perianal Pediatric Crohn’s Disease on Specific Carbohydrate Diet Monotherapy. JPGN Rep..

[41] Svolos V., Hansen R., Nichols B., Quince C., Ijaz U.Z., Papadopoulou R.T., Edwards C.A., Watson D., Alghamdi A., Brejnrod A. (2019). Treatment of Active Crohn’s Disease with an Ordinary Food-Based Diet That Replicates Exclusive Enteral Nutrition. Gastroenterology.

[42] Svolos V., Hansen R., Russell R., Gaya D.R., Paul S.J., Macdonald J., Nichols B., Papadopoulou R., Logan M., Mckirdy S. (2022). DOP68 CD-TREAT diet induces remission and improves quality of life in an open label trial in children and adults with active Crohn’s Disease. J. Crohn’s Colitis.

[43] Aharoni-Frutkoff Y., Plotkin L., Pollak D., Livovsky J., Focht G., Lev-Tzion R., Ledder O., Assa A., Yogev D., Orlanski-Meyer E. (2025). Whole Food Diet Induces Remission in Children and Young Adults with Mild to Moderate Crohn’s Disease and Is More Tolerable Than Exclusive Enteral Nutrition: A Randomized Controlled Trial. Gastroenterology.

[44] Plotkin L., Frutkoff Y.A., Shavit Z., Focht G., Livovsky J., Buchuk R., Lev-Zion R., Boneh R.S., Weiss B., Slae M. (2025). P1059 Fecal calprotectin response to Tasty&Healthy dietary intervention in asymptomatic children and young adults with biologically active Crohn’s disease: Results of the “TASTI-E” randomized controlled trial. J. Crohn’s Colitis.

[45] Plotkin L., Aharoni-Frutkoff Y., Shavit Z., Focht G., Livovsky J., Zion R.L., Ledder O., Kuint R.C., Zharkov E., Broide E. (2026). P0951 An exclusive whole-food Tasty&Healthy diet induces endoscopic and radiological healing in Crohn’s disease when used as maintenance treatment: Results from the MyTasty clinical trial. J. Crohn’s Colitis.

[46] Sarbagili-Shabat C., Albenberg L., Van Limbergen J., Pressman N., Otley A., Yaakov M., Wine E., Weiner D., Levine A. (2021). A Novel UC Exclusion Diet and Antibiotics for Treatment of Mild to Moderate Pediatric Ulcerative Colitis: A Prospective Open-Label Pilot Study. Nutrients.

[47] Chiba M., Tsuji T., Nakane K., Tsuda S., Ishii H., Ohno H., Watanabe K., Ito M., Komatsu M., Sugawara T. (2017). Induction with Infliximab and a Plant-Based Diet as First-Line (IPF) Therapy for Crohn Disease: A Single-Group Trial. Perm. J..

[48] Csiba-Tóth C., Dabóczi A., Howard M., Miller N.J., Clemens Z. (2016). Crohn’s disease successfully treated with the paleolithic ketogenic diet. Int. J. Case Rep. Images.

[49] El Amrousy D., Elashry H., Salamah A., Maher S., Abd-Elsalam S.M., Hasan S. (2022). Adherence to the Mediterranean Diet Improved Clinical Scores and Inflammatory Markers in Children with Active Inflammatory Bowel Disease: A Randomized Trial. J. Inflamm. Res..

[50] Sigall Boneh R., Assa A., Lev-Tzion R., Matar M., Shouval D., Shubeli C., Perets T.T., Chodick G., Shamir R. (2024). Adherence to the Mediterranean Diet Is Associated with Decreased Fecal Calprotectin Levels in Children with Crohn’s Disease in Clinical Remission under Biological Therapy. Dig. Dis..

[51] Clemente-Suárez V.J., Beltrán-Velasco A.I., Redondo-Flórez L., Martín-Rodríguez A., Tornero-Aguilera J.F. (2023). Global Impacts of Western Diet and Its Effects on Metabolism and Health: A Narrative Review. Nutrients.

[52] Temba G.S., Pecht T., Kullaya V.I., Vadaq N., Mosha M.V., Ulas T., Kanungo S., van Emst L., Bonaguro L., Schulte-Schrepping J. (2025). Immune and metabolic effects of African heritage diets versus Western diets in men: A randomized controlled trial. Nat. Med..

[53] Lewis J.D., Abreu M.T. (2017). Diet as a Trigger or Therapy for Inflammatory Bowel Diseases. Gastroenterology.

[54] Peters V., Spooren C.E.G.M., Pierik M.J., Weersma R.K., van Dullemen H.M., Festen E.A.M., Visschedijk M.C., Masclee A.A.M., Hendrix E.M.B., Almeida R.J. (2021). Dietary Intake Pattern is Associated with Occurrence of Flares in IBD Patients. J. Crohn’s Colitis.

[55] Li T., Qiu Y., Yang H.-S., Li M.-Y., Zhuang X.-J., Zhang S.-H., Feng R., Chen B.-L., He Y., Zeng Z.-R. (2020). Systematic review and meta-analysis: Association of a pre-illness Western dietary pattern with the risk of developing inflammatory bowel disease. J. Dig. Dis..

[56] Yan J., Wang L., Gu Y., Hou H., Liu T., Ding Y., Cao H. (2022). Dietary Patterns and Gut Microbiota Changes in Inflammatory Bowel Disease: Current Insights and Future Challenges. Nutrients.

[57] Agus A., Denizot J., Thévenot J., Martinez-Medina M., Massier S., Sauvanet P., Bernalier-Donadille A., Denis S., Hofman P., Bonnet R. (2016). Western diet induces a shift in microbiota composition enhancing susceptibility to Adherent-Invasive *E. coli* infection and intestinal inflammation. Sci. Rep..

[58] Breton J., Tu V., Tanes C., Wilson N., Quinn R., Kachelries K., Friedman E.S., Bittinger K., Baldassano R.N., Compher C. (2025). A pro-inflammatory diet is associated with growth and virulence of *Escherichia coli* in pediatric Crohn’s disease. J. Crohn’s Colitis.

[59] Peters V., Bolte L., Schuttert E.M., Andreu-Sánchez S., Dijkstra G., Weersma R., Campmans-Kuijpers M.E. (2022). Western and Carnivorous Dietary Patterns are Associated with Greater Likelihood of IBD Development in a Large Prospective Population-based Cohort. J. Crohn’s Colitis.

[60] Guo A., Ludvigsson J., Brantsæter A.L., Klingberg S., Östensson M., Størdal K., Mårild K. (2024). Early-life diet and risk of inflammatory bowel disease: A pooled study in two Scandinavian birth cohorts. Gut.

[61] Babaei A., Pourmotabbed A., Talebi S., Mehrabani S., Bagheri R., Ghoreishy S.M., Amirian P., Zarpoosh M., Mohammadi H., Kermani M.A.H. (2024). The association of ultra-processed food consumption with adult inflammatory bowel disease risk: A systematic review and dose-response meta-analysis of 4,035,694 participants. Nutr. Rev..

[62] Chen J., Wellens J., Kalla R., Fu T., Deng M., Zhang H., Yuan S., Wang X., Theodoratou E., Li X. (2023). Intake of Ultra-processed Foods Is Associated with an Increased Risk of Crohn’s Disease: A Cross-Sectional and Prospective Analysis of 187 154 Participants in the UK Biobank. J. Crohn’s Colitis.

[63] Narula N., Wong E.C.L., Dehghan M., Mente A., Rangarajan S., Lanas F., Lopez-Jaramillo P., Rohatgi P., Lakshmi P.V.M., Varma R.P. (2021). Association of ultra-processed food intake with risk of inflammatory bowel disease: Prospective cohort study. BMJ.

[64] Lo C.-H., Khandpur N., Rossato S.L., Lochhead P., Lopes E.W., Burke K.E., Richter J.M., Song M., Korat A.V.A., Sun Q. (2022). Ultra-processed Foods and Risk of Crohn’s Disease and Ulcerative Colitis: A Prospective Cohort Study. Clin. Gastroenterol. Hepatol..

[65] Jowett S.L., Seal C.J., Pearce M.S., Phillips E., Gregory W., Barton J.R., Welfare M.R. (2004). Influence of dietary factors on the clinical course of ulcerative colitis: A prospective cohort study. Gut.

[66] Dong C., Chan S.S.M., Jantchou P., Racine A., Oldenburg B., Weiderpass E., Heath A.K., Tong T.Y.N., Tjønneland A., Kyrø C. (2022). Meat Intake Is Associated with a Higher Risk of Ulcerative Colitis in a Large European Prospective Cohort Studyø. J. Crohn’s Colitis.

[67] Huang S., Yang K., Sun D., Pan L., Ma L., Li M., Shi W., Zong Y., Zhang Z., Li C. (2025). Red Meat Diet Exacerbates Colitis by Promoting the Accumulation of Myeloid Cells and Disrupting Gut Microbiota. Mol. Nutr. Food Res..

[68] Constantine-Cooke N., Gros B., Plevris N., Williams L.J., Jones G.-R., Kyle J., Kennedy N.A., Velasco-Pardo V., Rudge A., Alexander D. (2026). Associations between demographic, clinical and dietary factors and flares in inflammatory bowel disease: The PRognostic effect of Environmental factors in Crohn’s and Colitis (PREdiCCt) prospective cohort study. Gut.

[69] Chulenbayeva L., Jarmukhanov Z., Kaliyekova K., Kozhakhmetov S., Kushugulova A. (2025). Quantitative Alterations in Short-Chain Fatty Acids in Inflammatory Bowel Disease: A Systematic Review and Meta-Analysis. Biomolecules.

[70] Ozturk O., Celebi G., Duman U.G., Kupcuk E., Uyanik M., Sertoglu E. (2024). Short-chain fatty acid levels in stools of patients with inflammatory bowel disease are lower than those in healthy subjects. Eur. J. Gastroenterol. Hepatol..

[71] Saedi S., Derakhshan S., Sadeghi J., Hasani A., Khoshbaten M., Poortahmasebi V., Ahmadi S. (2025). A study on gut microbiota and short-chain fatty acids in patients with inflammatory bowel disease from northwest Iran. Lett. Appl. Microbiol..

[72] Li G., Lin J., Zhang C., Gao H., Lu H., Gao X., Zhu R., Li Z., Li M., Liu Z. (2021). Microbiota metabolite butyrate constrains neutrophil functions and ameliorates mucosal inflammation in inflammatory bowel disease. Gut Microbes.

[73] Ventura I., Chomon-García M., Tomás-Aguirre F., Palau-Ferré A., Legidos-García M.E., Murillo-Llorente M.T., Pérez-Bermejo M. (2024). Therapeutic and Immunologic Effects of Short-Chain Fatty Acids in Inflammatory Bowel Disease: A Systematic Review. Int. J. Mol. Sci..

[74] Fiorindi C., Russo E., Balocchini L., Amedei A., Giudici F. (2022). Inflammatory Bowel Disease and Customized Nutritional Intervention Focusing on Gut Microbiome Balance. Nutrients.

[75] Yu Y., Chen K.-C., Chen J. (2019). Exclusive enteral nutrition versus corticosteroids for treatment of pediatric Crohn’s disease: A meta-analysis. World J. Pediatr..

[76] Grover Z., Muir R., Lewindon P. (2014). Exclusive enteral nutrition induces early clinical, mucosal and transmural remission in paediatric Crohn’s disease. J. Gastroenterol..

[77] Grover Z., Burgess C., Muir R., Reilly C., Lewindon P.J. (2016). Early Mucosal Healing with Exclusive Enteral Nutrition is Associated with Improved Outcomes in Newly Diagnosed Children with Luminal Crohn’s disease. J. Crohn’s Colitis.

[78] Cohen-Dolev N., Sladek M., Hussey S., Turner D., Veres G., Koletzko S., de Carpi J.M., Staiano A., Shaoul R., Lionetti P. (2018). Differences in Outcomes Over Time with Exclusive Enteral Nutrition Compared with Steroids in Children with Mild to Moderate Crohn’s Disease: Results from the *GROWTH CD* Study. J. Crohn’s Colitis.

[79] Narula N., Dhillon A., Zhang D., Sherlock M.E., Tondeur M., Zachos M. (2018). Enteral nutritional therapy for induction of remission in Crohn’s disease. Cochrane Database Syst. Rev..

[80] Cuomo M., Carobbio A., Aloi M., Alvisi P., Banzato C., Bosa L., Bramuzzo M., Campanozzi A., Catassi G., D’aNtiga L. (2023). Induction of Remission with Exclusive Enteral Nutrition in Children with Crohn’s Disease: Determinants of Higher Adherence and Response. Inflamm. Bowel Dis..

[81] Logan M., Clark C.M., Ijaz U.Z., Gervais L., Duncan H., Garrick V., Curtis L., Buchanan E., Cardigan T., Armstrong L. (2019). The reduction of faecal calprotectin during exclusive enteral nutrition is lost rapidly after food re-introduction. Aliment. Pharmacol. Ther..

[82] Gkikas K., Logan M., Nichols B., Ijaz U.Z., Clark C.M., Svolos V., Gervais L., Duncan H., Garrick V., Curtis L. (2021). Dietary triggers of gut inflammation following exclusive enteral nutrition in children with Crohn’s disease: A pilot study. BMC Gastroenterol..

[83] Scarallo L., Lionetti P. (2021). Dietary Management in Pediatric Patients with Crohn’s Disease. Nutrients.

[84] Levine A., Wine E. (2013). Effects of enteral nutrition on Crohn’s disease: Clues to the impact of diet on disease pathogenesis. Inflamm. Bowel Dis..

[85] Matuszczyk M., Meglicka M., Wiernicka A., Jarzębicka D., Osiecki M., Kotkowicz-Szczur M., Kierkuś J. (2022). Effect of the Crohn’s Disease Exclusion Diet (CDED) on the Fecal Calprotectin Level in Children with Active Crohn’s Disease. J. Clin. Med..

[86] Dubner S.E., Shults J., Baldassano R.N., Zemel B.S., Thayu M., Burnham J.M., Herskovitz R.M., Howard K.M., Leonard M.B. (2009). Longitudinal assessment of bone density and structure in an incident cohort of children with Crohn’s disease. Gastroenterology.

[87] Braly K., Williamson N., Shaffer M.L., Lee D., Wahbeh G., Klein J., Giefer M., Suskind D.L. (2017). Nutritional Adequacy of the Specific Carbohydrate Diet in Pediatric Inflammatory Bowel Disease. J. Pediatr. Gastroenterol. Nutr..

[88] Suskind D.L., Lee D., Kim Y.-M., Wahbeh G., Singh N., Braly K., Nuding M., Nicora C.D., Purvine S.O., Lipton M.S. (2020). The Specific Carbohydrate Diet and Diet Modification as Induction Therapy for Pediatric Crohn’s Disease: A Randomized Diet Controlled Trial. Nutrients.

[89] Anon. Tasty&Heathy. https://tasty-healthy.com/the-diet/.

[90] Navas-López V.M., Herrador-López M., Torcuato-Rubio E., Sarbagili-Shabat C., Martín-Masot R. (2025). Quantitative and qualitative assessment of diet and its association with disease activity in pediatric ulcerative colitis. J. Pediatr. Gastroenterol. Nutr..

[91] Day A.S., Yao C.K., Costello S.P., Ruszkiewicz A., Andrews J.M., Gibson P.R., Bryant R.V. (2022). Therapeutic Potential of the 4 Strategies to SUlfide-REduction (4-SURE) Diet in Adults with Mild to Moderately Active Ulcerative Colitis: An Open-Label Feasibility Study. J. Nutr..

[92] Liu G.X.H., Day A.S. (2024). Plant-based Diets for Inflammatory Bowel Disease: What Is the Evidence?. Inflamm. Bowel Dis..

[93] Peng Z., Yi J., Liu X. (2022). A Low-FODMAP Diet Provides Benefits for Functional Gastrointestinal Symptoms but Not for Improving Stool Consistency and Mucosal Inflammation in IBD: A Systematic Review and Meta-Analysis. Nutrients.

[94] Olendzki B.C., Silverstein T.D., Persuitte G.M., Ma Y., Baldwin K.R., Cave D. (2014). An anti-inflammatory diet as treatment for inflammatory bowel disease: A case series report. Nutr. J..

[95] Olendzki B., Bucci V., Cawley C., Maserati R., McManus M., Olednzki E., Madziar C., Chiang D., Ward D.V., Pellish R. (2022). Dietary manipulation of the gut microbiome in inflammatory bowel disease patients: Pilot study. Gut Microbes.

[96] Alsharairi N.A. (2022). The Therapeutic Role of Short-Chain Fatty Acids Mediated Very Low-Calorie Ketogenic Diet–Gut Microbiota Relationships in Paediatric Inflammatory Bowel Diseases. Nutrients.

[97] Hashash J.G., Elkins J., Lewis J.D., Binion D.G. (2024). AGA Clinical Practice Update on Diet and Nutritional Therapies in Patients with Inflammatory Bowel Disease: Expert Review. Gastroenterology.

[98] Turpin W., Dong M., Sasson G., Garay J.A.R., Espin-Garcia O., Lee S.-H., Neustaeter A., Smith M.I., Leibovitzh H., Guttman D.S. (2022). Mediterranean-like Dietary Pattern Associations with Gut Microbiome Composition and Subclinical Gastrointestinal Inflammation. Gastroenterology.

[99] Godny L., Elial-Fatal S., Arrouasse J., Fischler T.S., Reshef L., Kutukov Y., Cohen S., Pfeffer-Gik T., Barkan R., Shakhman S. (2025). Mechanistic Implications of the Mediterranean Diet in Patients with Newly Diagnosed Crohn’s Disease: Multiomic Results from a Prospective Cohort. Gastroenterology.

[100] García-Mateo S., Martínez-Domínguez S.J., Gargallo-Puyuelo C.J., Gallego B., Alfambra E., Escuin M., García-Mateo S., López J., Gomollón F. (2025). Healthy Lifestyle Is a Protective Factor from Moderate and Severe Relapses and Steroid Use in Inflammatory Bowel Disease: A Prospective Cohort Study. Inflamm. Bowel Dis..

[101] Lewis J.D., Sandler R.S., Brotherton C., Brensinger C., Li H., Kappelman M.D., Daniel S.G., Bittinger K., Albenberg L., Valentine J.F. (2021). A Randomized Trial Comparing the Specific Carbohydrate Diet to a Mediterranean Diet in Adults with Crohn’s Disease. Gastroenterology.

[102] Bischoff S.C., Bager P., Escher J., Forbes A., Hébuterne X., Hvas C.L., Joly F., Klek S., Krznaric Z., Ockenga J. (2023). ESPEN guideline on Clinical Nutrition in inflammatory bowel disease. Clin. Nutr..

[103] Boneh R.S., Westoby C., Oseran I., Sarbagili-Shabat C., Albenberg L.G., Lionetti P., Navas-López V.M., Martín-De-Carpi J., Yanai H., Maharshak N. (2024). The Crohn’s Disease Exclusion Diet: A Comprehensive Review of Evidence, Implementation Strategies, Practical Guidance, and Future Directions. Inflamm. Bowel Dis..

[104] Zangara M.T., Bhesania N., Liu W., Cresci G.A.M., Kurowski J.A., McDonald C. (2020). Impact of Diet on Inflammatory Bowel Disease Symptoms: An Adolescent Viewpoint. Crohn’s Colitis 360.

[105] Gammanpila D., Collins T., Grover Z. (2026). Dietary Beliefs, Food Avoidance and Meal Skipping in Australian Children with Inflammatory Bowel Disease. J. Paediatr. Child Health.

[106] Boneh R.S., Banci E., Scarallo L., Herrador-López M., Halmos E.P., Shouval D.S., Wierdsma N., Sarbagili-Shabat C., Navas-López V.M., Martín-De-Carpi J. (2026). The Crohn’s Disease Exclusion Diet Across the Lifespan, Indications and the Globe: An Expert Review Towards Personalized Therapy in Crohn’s Disease. JCC Plus.

[107] Gatti S., Vallorani M., Quattrini S., Aloi M., Bramuzzo M., Felici E., Zuin G., Catassi G.N., Grazian F., Ciacchini B. (2024). Dietary habits in Italian children with inflammatory bowel disease: A case-control multicenter study. J. Pediatr. Gastroenterol. Nutr..

[108] Sigall-Boneh R., Organ S., Ghiboub M., Yanai H., Abramas L., Wierdsma N., Verburgt C.M., de Jonge W.J., Derikx J.P.M., Lewis J.D. (2026). Microbial predictors of sustained clinical remission following the Crohn’s disease exclusion diet in adults and microbiome shifts toward healthy paediatric controls. Crohn’s Colitis 360.

[109] Ghiboub M., Penny S., Verburgt C.M., Boneh R.S., Wine E., Cohen A., Dunn K.A., Pinto D.M., Benninga M.A., de Jonge W.J. (2022). Metabolome Changes with Diet-Induced Remission in Pediatric Crohn’s Disease. Gastroenterology.

[110] Ghiboub M., Boneh R.S., Sovran B., Wine E., Lefèvre A., Emond P., Verburgt C.M., Benninga M.A., de Jonge W.J., Van Limbergen J.E. (2023). Sustained Diet-Induced Remission in Pediatric Crohn’s Disease Is Associated with Kynurenine and Serotonin Pathways. Inflamm. Bowel Dis..

[111] Azulay A., Gotesdyner L., Aharoni-Frutkoff Y., Focht G., Talmor Y., Borenstein E., Plotkin L., Orlanski-Meyer E., Lev-Tzion R., Ledder O. (2026). Multi-omics–based machine learning model predicts response and guides treatment in Crohn disease: A case study in nutritional therapy. Inflamm. Bowel Dis..

